# Functional connectivity of semantic and default mode networks during narrative comprehension

**DOI:** 10.1093/cercor/bhaf289

**Published:** 2025-11-04

**Authors:** Melissa Thye, Junhua Ding, Paul Hoffman, Daniel Mirman

**Affiliations:** Department of Psychology, University of Edinburgh, 7 George Square, Edinburgh EH8 9JZ, United Kingdom; State Key Laboratory of Cognitive Science and Mental Health, Institute of Psychology, Chinese Academy of Sciences, 16 Lincui Road, Beijing 100101, China; Department of Psychology, University of Edinburgh, 7 George Square, Edinburgh EH8 9JZ, United Kingdom; Department of Psychology, University of Edinburgh, 7 George Square, Edinburgh EH8 9JZ, United Kingdom

**Keywords:** default mode network, functional MRI, movie-viewing, narrative comprehension, semantic cognition

## Abstract

Understanding narratives requires at least transient access to the semantic system, to decode incoming content, and prolonged access to the default mode network to maintain and manipulate the narrative model. Subregions within the integrative semantic hubs in bilateral anterior temporal lobe appear differentially sensitive to the need to rapidly decode external input (exogenous processing) versus reflecting on context stored in the narrative model (endogenous processing). The latter is most consistently reported in the middle temporal gyrus portion of the hub, suggesting that this region serves as a critical hinge point, dynamically interacting with the default mode network to facilitate endogenous processing. The present study investigated this by characterizing the functional connectivity profiles of anterior temporal lobe subregions during movie-viewing and examining content-evoked changes in these profiles. Compared to other anterior temporal lobe subregions, middle temporal gyrus was more functionally connected to the default mode network, and these connections were strengthened during moments with limited incoming information, providing viewers with a chance to reflect on the content. Rather than being functionally distinct networks, the semantic and default mode systems dynamically interact to facilitate reflection or endogenous semantic processing. Future work should further characterize how neural systems dynamically shift from integrated to segregated states in response to everyday processing demands.

## Introduction

Convergent neuropsychological and neuroimaging evidence points to the existence of a distributed semantic system that represents lexical and object concepts and their relations with one another (Binder et al. 2009; McRae and Jones 2013; Lambon Ralph et al. 2017). This semantic system plays an active role in processing narratives and natural language, but its specific contributions are unclear. More than decoding the individual conceptual units that are used to the tell the story, the semantic system must retrieve and help organize meaning to form a coherent understanding of the narrative. Key to ongoing comprehension of narrative input is the construction and maintenance of a situation model in which the narrative context is stored (Zwaan and Radvansky 1998; Yarkoni et al. 2008). This is primarily attributed to the default mode network (DMN), and recent evidence hints at how the semantic system contributes to this process.

Although semantic processing relies on a distributed set of regions, many studies indicate a critical role for the bilateral anterior temporal lobes (ATLs), which appear to act as integrative semantic hub regions (Patterson et al. 2007; Lambon Ralph et al. 2017). Subregions within these core semantic hubs are differentially engaged by narrative content. In particular, the dorsolateral part, corresponding to superior temporal gyrus (STG), and the ventral part, corresponding to fusiform gyrus (FG), appear to be sensitive to the amount and informativeness of new narrative content. In contrast, a portion of the ventrolateral part—centered on the middle temporal gyrus (MTG)—appears to be particularly engaged by social and pragmatic content that relies on knowledge of the preceding narrative for interpretation (Thye et al. 2024a). Although ventrolateral ATL includes both the inferior and MTG, these effects were primarily observed in MTG in our previous work (Thye et al. 2024a). Given the general role of the MTG portion of the ATL hub in wide-ranging, non-social semantic tasks (Lambon Ralph et al. 2017), it is unlikely that its response during narrative comprehension is driven by the socialness of the input per se. Instead, its apparent preference for social content may arise because significant moments in a narrative are often social in nature. Further, pragmatic content requires the situation model for correct interpretation; that is, the narrative context is needed for interpreting this non-literal input. This argument is in line with prior theoretical (Binney and Ramsey 2020) and empirical work (Balgova et al. 2022; Rouse et al. 2024) that suggests that the semantic system, particularly in ATL, carries out domain-general cognitive processes that also lend themselves well to socio-cognitive processing. Therefore, increased activation in MTG has been interpreted as reflecting engagement in semantic cognition that is driven by the situation model, termed ‘*endogenous semantic processing*’ to distinguish it from ‘*exogenous semantic processing*’ that is driven by external input and associated with engagement of FG and STG.

By exogenous semantic processing, we refer to the rapid decoding and comprehension of incoming perceptual/language input. In contrast, we have proposed that endogenous semantic processing occurs when the narrative requires or allows accessing and reflecting on context stored in the situation model (Thye et al. 2023, 2024a, 2024b). Highly semantic (informative) incoming content engages different ATL subregions compared to content that requires reflection on the narrative context, suggesting that exogenous and endogenous semantic processing place different demands on both the semantic system and the narrative system within the DMN. This account is further bolstered by the structural connections underlying ATL subregions. The MTG subregion has more widespread structural connections than the other ATL subregions, including connections to the core regions within the DMN: angular gyrus, dorsomedial prefrontal cortex (dmPFC), posterior cingulate cortex, and precuneus (Fan et al. 2014; Sasaki et al. 2023). In contrast, STG receives inputs from earlier auditory processing regions and FG is strongly connected with the occipito-temporal visual processing stream (Binney et al. 2012). These differing patterns of connectivity suggest a distinction between semantic processing of sensory inputs vs. semantic contribution to internally-represented situation models.

**Fig. 1:**
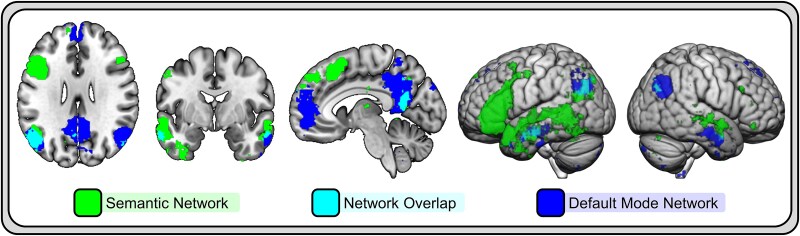
Overlap between the semantic and default mode networks. Networks maps generated using Neurosynth (https://neurosynth.org/) showing regions associated with the terms “semantic” and “default mode”.

Although different functional roles are ascribed to these systems, a complicating factor in delineating their boundaries is considerable cross-network overlap between the semantic system and DMN (Fig. 1). Regions within the DMN are consistently reported in meta-analyses of task-based activation studies of the semantic system (Binder et al. 2009). Conversely, definitions of the DMN often include a subsystem or component that is comprised of semantic network regions, including portions of the ATL (Andrews-Hanna et al. 2014). Despite this overlap, the systems are separable (Wang et al. 2021). In general, core DMN regions deactivate in response to attention demanding, externally oriented semantic tasks (Humphreys et al. 2015; Shao et al. 2024), whereas activation increases in semantic network regions during these tasks. Further, broad definitions of the semantic network that include DMN regions can be decomposed into a tripartite network structure, in which the DMN emerges as a separable module (Xu et al. 2016). This module does not include ATL, suggesting different functional roles. However, in this work, left MTG serves as a hub connecting the DMN to a perisylvian semantic network.

Rather than focusing on overlapping versus distinct regions, it may be more productive to characterize how the semantic and default mode networks dynamically interact. Instead of a highly modularized system of networks specialized for carrying out discrete tasks, brain networks have been shown to fluctuate between segregated and integrated states in response to external demands. Such task-evoked reconfiguration of networks relates to improved cognitive performance (Shine et al. 2016). In the narrative context, variations in content and processing demands over the course of a narrative may drive variable network integration to facilitate successful comprehension and even enjoyment. For example, large amounts of verbal input might drive stronger integration of language and semantic systems, and disengagement from the DMN, but ambiguous input that requires the situation model for interpretation may drive stronger integration between the semantic and default mode networks. Prior research examining semantic system involvement in narrative processing has predominately used activation-based analyses, but the underlying claims about the functional connectivity between these systems have rarely been tested.

The primary aim of the present study was to characterize the stable and content-evoked functional connectivity profiles of the ATL subregions during movie-viewing. This was accomplished by first conducting whole-brain, exploratory functional connectivity analyses of a large fMRI movie-viewing dataset, with four bilateral ATL subregions: anterior fusiform, anterior inferior temporal gyrus (ITG), anterior MTG, and anterior STG. This was followed by focused, confirmatory analyses examining ATL subregion functional connectivity with core DMN regions. We anticipated that MTG would have strongest connections to the DMN. Finally, to examine content-evoked network reconfiguration, the dynamic functional connectivity of the same ATL subregions was covaried with continuous measures of the semantic and social narrative content. Based on the endogenous semantic processing account, we expected periods of low on-screen semantic content to capture moments when the viewer is accessing and reflecting on their situation model. This should lead to an increase in the functional connections between MTG and the DMN. Conversely, we expected that periods of high semantic content would be associated with increased functional connections between STG and fusiform and the rest of the semantic system, given their proposed role in exogenous semantic processing. A measure of social content was included in the analyses in order to test an alternative to the endogenous semantic processing account: that MTG is specialized for social processing. If this is the case, then we might observe (i) strong, static functional connections between the MTG and the DMN and no content-evoked changes in these connections because MTG is part of a robustly functionally connected social network or (ii) increased connectivity to DMN regions when social, but not semantic content, is high. This is of particular importance because many of the regions within the DMN are ascribed social functions (Mars et al. 2012; Monticelli et al. 2021). Including social content allows for testing whether MTG connectivity is determined by social processing or by the occurrence of low-semantic moments which provide the viewer with the opportunity to engage in endogenous semantic processing.

## Materials and methods

### Dataset

The present study used preprocessed data from version 2.0.0 of the Naturalistic Neuroimaging Database (NNDb; https://openneuro.org/datasets/ds002837/versions/2.0.0), a publicly available dataset in which participants (*n* = 86; mean age = 26.81) watched one of ten full length movies while undergoing fMRI. See the publication describing the dataset for a detailed overview of the experimental procedures, including how the data were collected and preprocessed (Aliko et al. 2020). The research complies with all relevant ethical regulations. The project from which the data are derived was approved by the ethics committee of University College London. Participants provided written informed consent to take part in the study and have their anonymised data shared.

### Narrative annotations

Semantic and social event ratings that were generated and described in a prior study (Thye et al. 2024b) were used for the dynamic functional connectivity analyses. Briefly, each movie was segmented into discrete events based on principles of event segmentation, namely that event boundaries occur when a sequence feels disconnected, unexpected, unrelated, or discontinuous from the previous sequence (Zacks et al. 2011; Buchsbaum et al. 2015; Güler et al. 2025). The full movie was used for the static functional connectivity analyses, but the closing credits were removed prior to the dynamic functional connectivity analyses given that these events minimally contribute to the narrative. Excluding the closing credits, the number of events per movie ranged from 238 to 421 (Mdn = 363).

The semantic and social content of each event was rated on a scale from 1 (low) to 10 (high) following a detailed protocol (https://osf.io/38ax7). Semantic content was defined as narrative exposition in which information is presented linguistically through spoken language (by a character or narrator) or in writing (such as text about the movie, timescale, or characters or any text presented during an event). Events in which new semantic information was presented were coded as more semantic relative to events with semantic content that was already known to the viewer. This criterion was included because events with novel information are more informative and require greater semantic processing relative to the moments in which the information is consistent with what has already been processed. Low semantic events would have minimal to no written or spoken exchange of new information, such as an action sequence. Social content was defined by the presence of more than one person or character, even if inanimate or off-screen. Any event that conveys information about the characters in the movie and their relationships with other characters was considered social. The relative degree of sociality depended on the type, duration, and significance of the interaction within the event. Although an event may be both highly social and emotional, an event did not have to be emotionally intense in order to be considered social. Similarly, social and semantic content were coded independently as an event can be both highly (or weakly) social and semantic. The highest ratings (ie 9 or 10) were reserved for events in which the primary purpose of the scene was to convey semantic or social information. Importantly, a single event could not receive a 9 or 10 for both semantic and social content because the primary purpose had to be coded as either semantic or social. These ratings are described in greater detail in the prior publication (Thye et al. 2024b).

At least two independent coders rated the semantic and social content of each event and wrote a brief description of what occurred during the event (see Table 1 for examples). Inter-rater reliability was assessed separately for the semantic and social scores for the coders of each movie using Krippendorff’s alpha reliability coefficient. When the inter-rater reliability fell below 0.75, the coder who rated all movies identified which events were poorly aligned, rewatched the event, and made a revised consensus rating based on the content of the event and the notes of the other coder.

**Table 1:** Example movie event annotations.

**Event**	**Semantic**	**Social**	**Event notes**
	**R1 (R2)**		
402	1 (1)	3 (2)	Red on the bus (mirroring Brooks when he was released)
403	4 (3)	1 (2)	Red being shown into his apartment—it’s where Brooks lived and carved his name
404	2 (2)	3 (3)	Red at the grocery store working as a bagger
405	3 (4)	5 (4)	Red asks his boss for a restroom break—the boss tells him to just go to the bathroom when he needs to
406	3 (5)	1 (1)	Red in the bathroom narrating how he’s had to ask for permission for 40 years
407	6 (6)	1 (1)	Red narrating that he can’t make it on the outside and all he does is look for ways to break parole so maybe they’d send him back
408	7 (7)	1 (1)	Red narrating that he wanted to be in a place where things make sense with no fear but he made a promise to Andy
409	2 (3)	3 (3)	Red being driven in car to a field
410	3 (1)	1 (1)	Red walking down the road to the location Andy told him about
411	1 (1)	1 (1)	Red walking along the wall Andy told him about
412	1 (1)	1 (1)	Red finding the rock
413	1 (1)	1 (1)	Red finds a small container under the rock
414	2 (1)	1 (1)	Red opens the container—he finds an envelope of money and a letter
415	7 (6)	3 (1)	Red opens the letter and we hear Andy read it—Andy tells him to join him and reminds him to have hope

*Note.* Representative events taken from “The Shawshank Redemption”. For the Semantic and Social ratings, the primary coder ratings (R1) are shown as well as ratings from a secondary coder (R2) which are shown in parentheses. Event Notes are from the primary coder (R1). Event Number refers to the Minor Event Number.

### Regions of interest

**Fig. 2:**
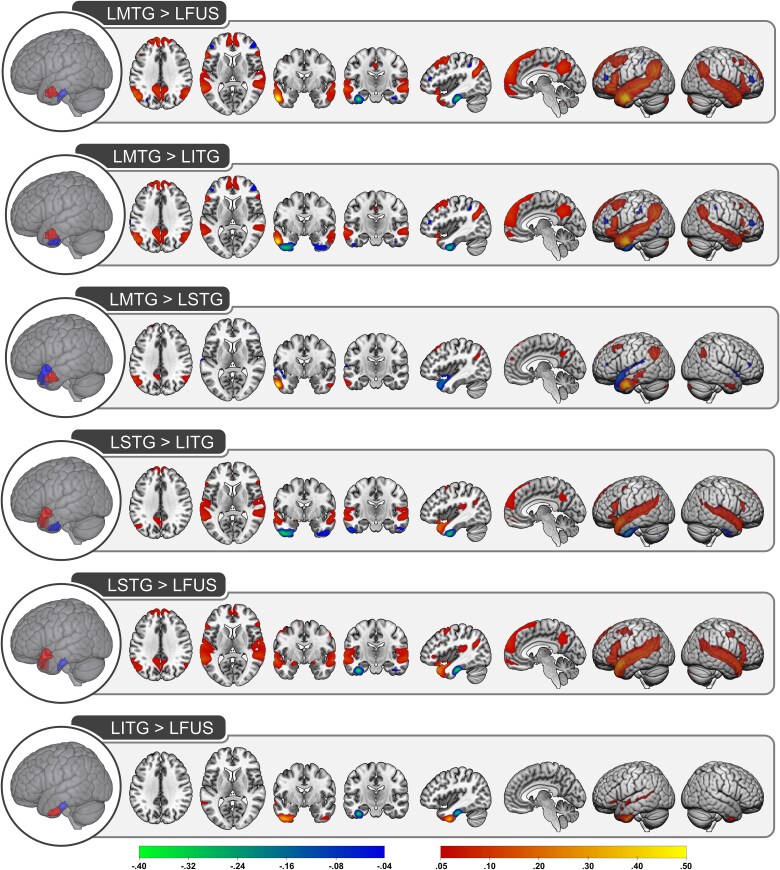
Pairwise comparisons of the functional connections between left hemisphere ATL subregions. The corrected Z-statistical values are shown for each pairwise comparisons. Positive values (indicated with warm colors increasing from red to yellow) reflect connections that are greater for the region shown in red relative to the region shown in blue. Negative values (indicated with cool colors increasing from blue to green) reflect connections that are greater for the region shown in blue relative to the region shown in red. LMTG, left middle temporal gyrus; LFUS, left fusiform; LITG, left inferior temporal gyrus; LSTG, left superior temporal gyrus.

**Fig. 3:**
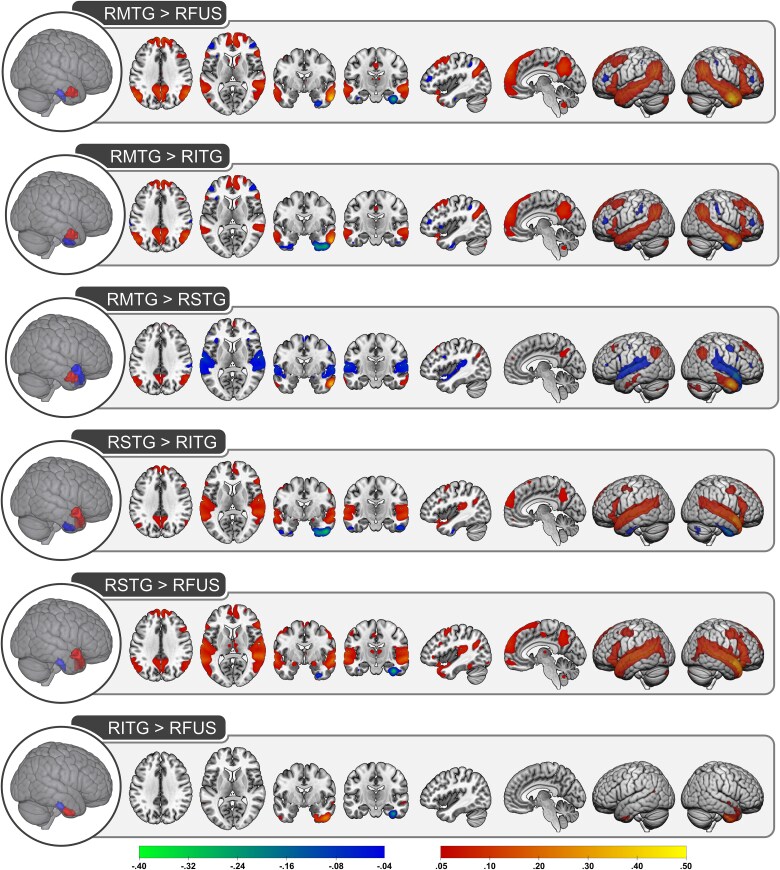
Pairwise comparisons of the functional connections between right hemisphere ATL subregions. The corrected Z-statistical values are shown for each pairwise comparisons. Positive values (indicated with warm colors increasing from red to yellow) reflect connections that are greater for the region shown in red relative to the region shown in blue. Negative values (indicated with cool colors increasing from blue to green) reflect connections that are greater for the region shown in blue relative to the region shown in red. RMTG, right middle temporal gyrus; RFUS, right fusiform; RITG, right inferior temporal gyrus; RSTG, right superior temporal gyrus.

Bilateral ATL subregions (shown in Fig. 2 and 3) were defined using the two most anterior segments from a prior study which parcellated the temporal gyri into anterior to posterior segments (Hoffman and Lambon Ralph 2018). They covered approximately the anterior one-third of each temporal gyrus. The DMN regions were defined using the bilateral precuneus, posterior division of the cingulate gyrus, and angular gyri within the Harvard Oxford atlas and dmPFC was defined using the left and right medial portions of the superior frontal gyrus (A10m) within the Brainnetome atlas (Fan et al. 2016). Bilateral V1 ROIs were included as a hypothesis-neutral control region. These ROIs (shown in Supplemental Fig. 4) were derived from a probabilistic atlas of retinotopic regions within the visual cortex (Wang et al. 2015). The dorsal and ventral V1 were first thresholded by 25% and then combined separately for each hemisphere.

### Functional connectivity analyses

Functional connectivity analyses were done using the Data Processing and Analysis for Brain Imaging (DPABI) toolbox (Yan et al. 2016) and SPM12 (https://fil.ion.ucl.ac.uk/spm) in MATLAB R2023b (The MathWorks, Inc 2023).

#### Pairwise contrasts of ATL regions

Each ATL region was used as a seed in separate functional connectivity analyses that produced whole brain statistical maps reflecting the voxelwise correlations with the average timecourse of the seed region. Using the Fisher *r* to *z* transform, these correlation maps were transformed to normalized Z-maps which were used for subsequent analyses.

The normalized subject-level functional connectivity Z-maps for the four ATL subregions (*n* = 86 for each region) were entered into a one-way repeated measures ANOVA with four levels (fusiform, ITG, MTG, and STG) performed at each voxel within a group gray matter mask. Pairwise comparisons (*n* = 6) between the ATL subregions were extracted from the omnibus model, correcting for multiple comparisons using Bonferroni correction. This was done separately for each hemisphere. The resulting *p* maps were converted to *Z* maps according to the normal inverse cumulative distribution function (norminv), with the sign of group mean differences applied. These *Z* maps were further thresholded to account for the number of voxelwise comparisons (i) using a threshold of *P* < 0.001 and a cluster extent of 30 voxels and (ii) removing values <10% of the maximum (for positive associations, 0.05) or minimum value for negative associations, −.04). This analysis was done for the left and right hemisphere ROIs separately (ie two separate ANOVAs) because we were not interested in direct comparisons between left versus right hemisphere ATL subregions.

Although we were primarily interested in the pairwise differences between the ATL subregions, we also conducted one-sample *t-*tests for each subregion using the normalized subject-level functional connectivity maps (*n* = 86) as inputs. The resulting unthresholded *t* statistic maps for each region are provided in Supplemental Fig. 1 (left hemisphere subregions) and Supplemental Fig. 2 (right hemisphere subregions).

#### Connectivity between ATL and DMN regions

**Fig. 4:**
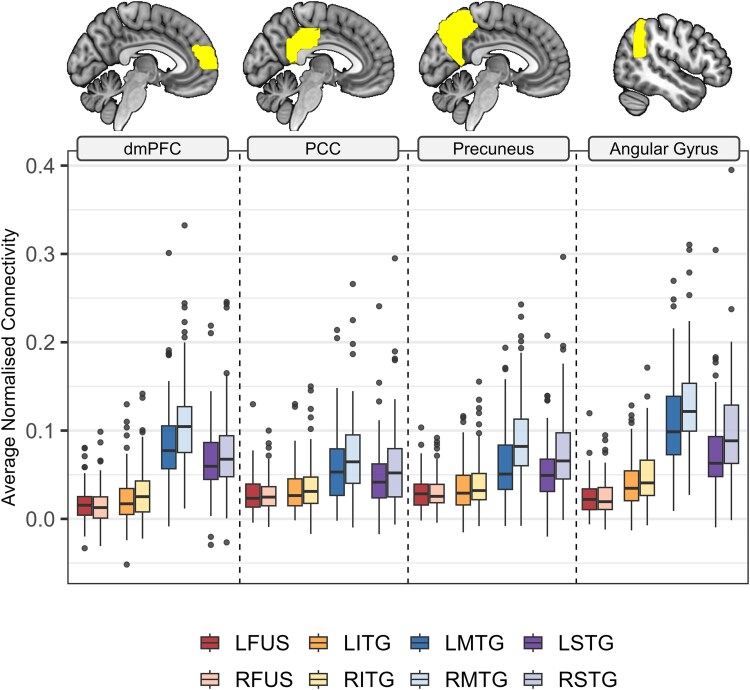
Average functional connectivity values in DMN regions. Each panel shows the functional connectivity z-score values between the ATL subregions (boxplots) and regions within the DMN (pictured in yellow at the top of each panel). From left to right: dmPFC, dorsomedial prefrontal cortex; PCC, posterior cingulate cortex; precuneus; bilateral angular gyrus. The extracted values reflect the strength of the functional connections between each DMN region and the left (more saturated) and right (less saturated) ATL subregions shown in each panel: FUS, fusiform (red); ITG, inferior temporal gyrus (orange); MTG, middle temporal gyrus (blue); STG, superior temporal gyrus (purple).

In order to examine the strength of the functional connections between each ATL subregion and regions within the DMN, we extracted the mean normalized functional connectivity value within each DMN region (defined above and shown in Fig. 4) from the subject-level connectivity maps. These values were used as the dependent variable in a mixed effects model with fixed effects of hemisphere (left, right), ATL region (fusiform, ITG, MTG, and STG), DMN region (dmPFC, PCC, precuneus, angular gyri), and the ATL-by-DMN region interaction with random intercepts for subject and random slopes for the effects of ATL region, DMN region, and hemisphere within subjects. This random effects structure accounts for subject-level variability that is unrelated to the outcome variable by allowing for differences in the baseline degree of functional connectivity between ATL or DMN regions and by allowing the effects of region (ATL or DMN) and hemisphere to vary across subjects. From this omnibus model we extracted pairwise comparisons (*n* = 6), correcting for multiple comparisons using Tukey’s method. Data were analyzed using the lme4 package (version 1.1–35.5) (Bates et al. 2015) in R (version 4.4.0) (R Core Team 2023). Model parameter *P-*values were obtained using the Satterthwaite method for estimating degrees of freedom via the lmerTest package (version 3.1–3) (Kuznetsova et al. 2017).

### Dynamic functional connectivity analyses

To investigate dynamic changes in functional connectivity driven by the content of the movie, the dynamic connectivity of each ATL subregion was separately estimated by calculating the functional connectivity between each ATL seed region and the whole brain using a 60s Hamming window function, sliding every 4 s (sensitivity analyses using 30s and 120s Hamming windows produced the same pattern of results). We calculated the dynamic functional connectivity of a control region, bilateral V1, in an analogous way to ensure that any observed content-evoked fluctuations were not driven by the low-level features or temporal autocorrelation inherent to the movie. For each of these regions, we then examined the correlations between the dynamic functional connectivity timecourses and semantic, social, or hypothesis-neutral content. The volumes corresponding to the closing credits were removed prior to analysis.

For each movie, the narrative semantic and social scores as well as two hypothesis-neutral measures were included in analysis. The first hypothesis-neutral measure was a vector of scrambled scores. The second hypothesis-neutral measure was edge density, which was calculated from each frame of the movie after conversion to grayscale. A greater number of edges within a frame indexes higher visual complexity, which may drive changes in the functional connections of V1, but should not drive changes in the functional connections of the ATL subregions. The hypothesis-neutral measures were included as null control variables that had no meaningful relationship with the narrative content to validate the robustness of the analytic approach.

The semantic, social, and hypothesis-neutral measures were smoothed in an analogous manner to the functional data with a 60s Hamming window function, sliding every 4 s. The smoothed scores were separately used as covariates to identify whether changes in the dynamic functional connectivity of each ATL subregion or V1 covaried with fluctuations in each content type.

The Pearson correlation coefficient between the whole-brain dynamic functional connectivity of each region and semantic, social, or hypothesis-neutral scores was calculated and converted to a Z statistical map using the Fisher *r* to *z* transform. A one-sample *t*-test was used to aggregate the subject-level results. For each hemisphere, the resulting group-level statistical maps for the ATL subregions (*n* = 4) were thresholded using a Bonferroni corrected threshold of *P* < 0.00025 and a cluster extent of 30. The group-level statistical maps for the V1 subregions (*n* = 2) were thresholded using a Bonferroni corrected threshold of *P* < 0.0005 and a cluster extent of 30.

Analysis code, movie annotations, and results files are available on OSF (https://osf.io/76hfv).

## Results

### Functional connectivity

#### Pairwise contrasts of ATL regions

The pairwise comparisons between ATL subregions are shown separately for each hemisphere in Fig. 2 and 3 and coordinate information is provided in Supplemental Table 1.

As expected, the pairwise contrasts consistently identified differences in functional connectivity such that, compared to other subregions, connectivity was greater within and proximal to each subregion as well as within the contralateral subregion. The results were similar across the left and right hemisphere, suggesting that left and right ATLs have analogous functional connections.

Critically, the connectivity profiles differed between the ATL subregions. Anterior fusiform and ITG tended to have weaker functional connections compared to the other subregions, which may be driven by signal drop-out in ventral ATL (Ojemann et al. 1997). The exception was increased connectivity between fusiform/ITG (compared to MTG) and bilateral inferior frontal gyrus and a portion of inferior parietal lobule. Differences between ITG and fusiform were predominately limited to each subregion, but ITG had stronger functional connections to STG and left ITG had greater connections to the orbital portion of inferior frontal gyrus.

When contrasted with the other subregions, both MTG and STG had stronger functional connections to regions within the DMN (precuneus, angular gyrus, and dmPFC) and the canonical language network (STG, middle frontal gyrus). Direct comparison of the functional connections of MTG and STG suggest an approximate division along these network boundaries such that the language regions are more strongly connected to STG whereas MTG has stronger connections to the DMN.

#### Connectivity between ATL and DMN regions

These functional connections were further investigated by extracting mean connectivity from DMN regions. The results are presented in Fig. 4. The predictor effect plot extracted from the mixed effects model showing the model fit values for each DMN region of interest and ATL subregion is provided as Supplemental Fig. 3. Connectivity was consistent across the DMN regions, with clear differences between ATL regions (collapsing across hemisphere): connectivity was greater for MTG versus STG (*Est.* = 0.02, *SD* = 0.002, *t*(87) = 10.21, *P* < 0.001), STG versus ITG (*Est.* = 0.03, *SD* = 0.003, *t*(87) = 11.40, *P* < 0.001), and ITG versus FUS (*Est.* = 0.01, *SD* = 0.002, *t*(87) = 7.04, *P* < 0.001). All extracted pairwise comparisons are reported in Supplemental Table 2. The connectivity profiles between each ATL subregion and the DMN was graded and, topographically, followed a U-shape pattern such that connectivity was generally lowest for bilateral fusiform and inferior temporal gyri, highest for middle temporal gyri, and somewhat lower for superior temporal gyri. Although the pattern of results was the same, functional connectivity with the DMN regions was generally stronger for right ATL subregions (*Est.* = 0.01, *SD* = 0.001, *t*(87) = 7.67, *P* < 0.001).

### Dynamic functional connectivity

#### Semantic content

**Fig. 5:**
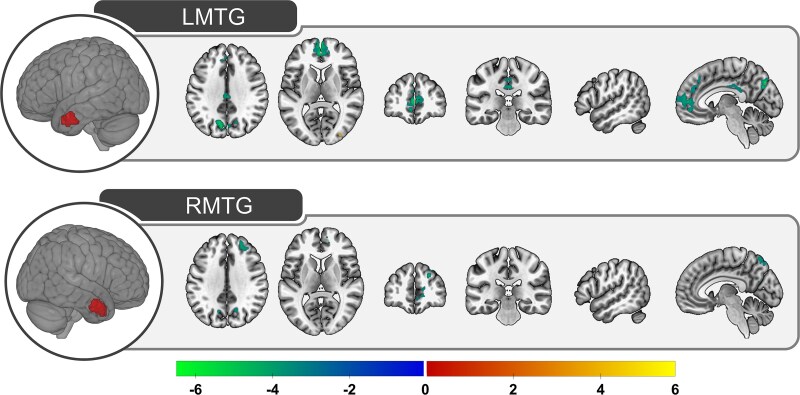
Dynamic functional connectivity associations with semantic content. The thresholded *t-*statistic images after conducting a one-sample *t-*test using the normalized dynamic functional connectivity maps for each subregion (*n* = 86) as inputs. LMTG, left middle temporal gyrus; RMTG, right middle temporal gyrus. The strength of negative associations with semantic content is indicated in cool colors, ranging from blue to green. The strength of positive associations with semantic content is indicated in warm colors, ranging from red to yellow. Results are displayed on the same slices (axial: 34, 8; coronal: 46, −28; sagittal: 52, −5).

The functional connectivity profiles of bilateral MTG covaried with fluctuations in semantic content. These results are presented in Fig. 5 and coordinate information is provided in Table 2. Functional connectivity of STG, ITG, or FUS did not covary with semantic content.

**Table 2:** Semantic content coordinate table.

**Region**	**Association**	**Cluster size**	**Hem**	**Peak voxel**	**MNI coordinates**		
					**x**	**y**	**z**
LMTG	Positive	93	R	Middle occipital gyrus	28.5	−88.5	16.5
LMTG	Negative	400	R	Medial frontal gyrus	7.5	55.5	4.5
		160	L	Precuneus	−4.5	−76.5	37.5
		66	R	Cingulate gyrus	4.5	−25.5	31.5
RMTG	Negative	187	L	Precuneus	−10.5	−76.5	46.5
		63	R	Superior frontal gyrus	22.5	43.5	31.5
		30	R	Medial frontal gyrus	10.5	55.5	4.5

*Note.* Hem, hemisphere; L, left; R, right; LMTG, left middle temporal gyrus; RMTG, right middle temporal gyrus.

There was a positive association between functional connectivity in left MTG and semantic content in a small cluster in right middle occipital gyrus. This means that during periods of high semantic content in the movie, the functional connections between left MTG and this region increased. No positive associations were observed for right MTG.

**Fig. 6:**
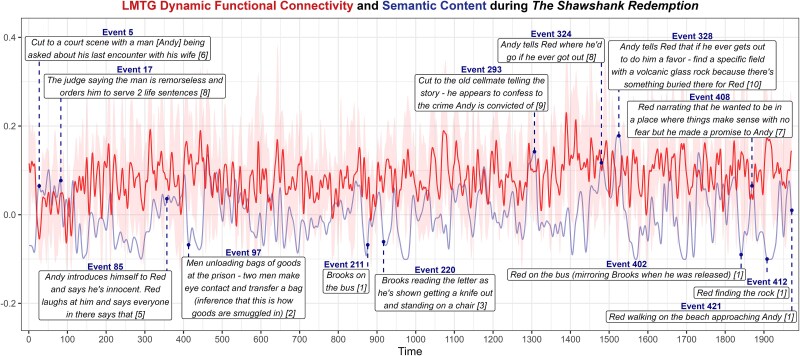
Illustrative example of the negative correlation between LMTG dynamic functional connectivity and semantic scores. The dynamic functional connectivity timecourse (red) extracted from the midline network of regions with negative associations with semantic content (blue) averaged for participants (*n* = 5) who watched “The Shawshank Redemption”. The event numbers and annotations are connected to each approximate event location with a dashed line. Semantic content scores for each event are provided in brackets after the event description.

There were also negative associations such that an increase in semantic content was associated with a decrease in the strength of connections with bilateral MTG. For left MTG, this included medial frontal gyrus (including dmPFC), posterior cingulate, and precuneus. For right MTG, this included precuneus, right superior frontal gyrus, and right medial frontal gyrus. To better understand the negative association between left MTG dynamic connectivity and semantic content, the timecourses from the cluster of midline regions showing a negative association were extracted and plotted alongside the smoothed semantic scores for one of the movies (Fig. 6). Highly informative events (events 5, 17, 293, 324, 328, and 408) were associated with reduced connections between left MTG and these midline regions. Conversely, events with minimal semantic content in which the narrator (and audience) make inferences or reflect on prior narrative content (85, 97, 211, 220, 402, 412, 421) are associated with increased connectivity between left MTG and these midline regions. This further illustrates that connectivity between the DMN and left MTG tended to be stronger during periods of low extrinsic semantic content.

#### Social content

**Fig. 7:**
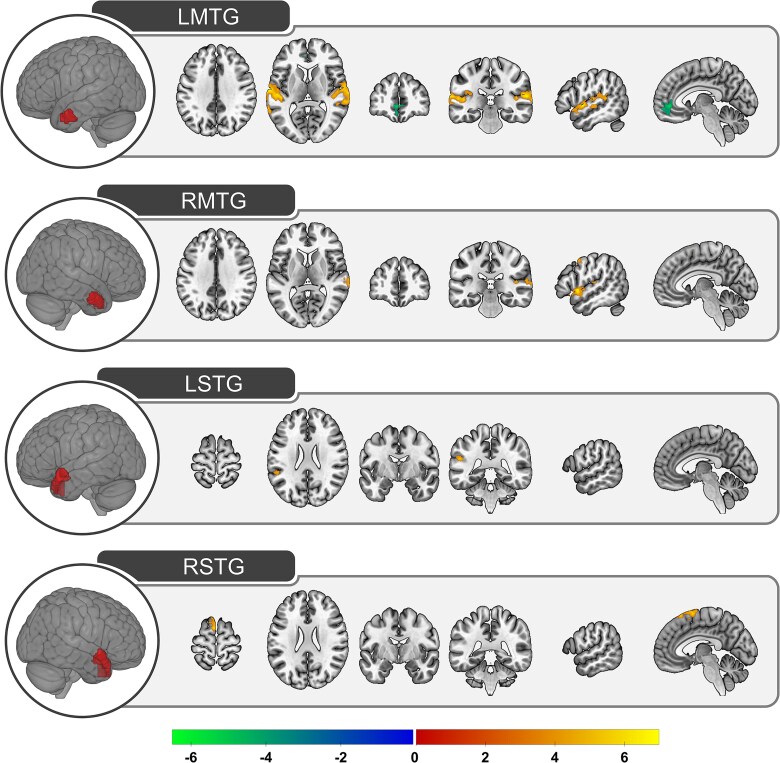
Dynamic functional connectivity associations with social content. The thresholded *t-*statistic images after conducting a one-sample *t-*test using the normalized dynamic functional connectivity maps for each subregion (*n* = 86) as inputs. LMTG, left middle temporal gyrus; RMTG, right middle temporal gyrus; LSTG, left superior temporal gyrus; RSTG, right superior temporal gyrus. The strength of negative associations with semantic content is indicated in cool colors, ranging from blue to green. The strength of positive associations with semantic content is indicated in warm colors, ranging from red to yellow. Results are displayed on the same slices for LMTG and RMTG (axial: 34, 8; coronal: 46, −28; sagittal: 52, −5) and different slices for LSTG and RSTG (axial: 68, 24; coronal: −6, −40; sagittal: 58, −4).

The functional connectivity profiles of bilateral MTG and STG covaried with fluctuations in social content. Functional connectivity did not vary with social content in any of the other ATL subregions. The results are presented in Fig. 7 and coordinate information is provided in Table 3.

**Table 3:** Social content coordinate table.

**Region**	**Association**	**Cluster size**	**Hem**	**Peak voxel**	**MNI coordinates**		
					**x**	**y**	**z**
LMTG	Positive	575	L	Superior temporal gyrus	−61.5	−31.5	13.5
		492	R	Superior temporal gyrus	61.5	−28.5	13.5
LMTG	Negative	101	L	Superior frontal gyrus	−7.5	55.5	1.5
RMTG	Positive	87	R	Superior temporal gyrus	52.5	7.5	−1.5
		82	R	Superior temporal gyrus	64.5	−25.5	13.5
		30	R	Precentral gyrus	52.5	7.5	49.5
		30	R	Supplementary motor area	4.5	1.5	70.5
LSTG	Positive	31	L	Superior temporal gyrus	−49.5	−37.5	19.5
RSTG	Positive	72	L	Supplementary motor area	−4.5	25.5	64.5

*Note.* Hem, hemisphere; L, left; R, right; LMTG, left middle temporal gyrus; RMTG, right middle temporal gyrus; LSTG, left superior temporal gyrus; RSTG, right superior temporal gyrus.

In left MTG, an increase in social content was associated with an increase in the strength of connections with bilateral superior temporal gyri whereas a decrease in social content was associated with an increase in the strength of connections to superior medial frontal gyrus. This cluster included dmPFC, but primarily overlapped with ventromedial prefrontal cortex.

In right MTG, a similar pattern was observed—an increase in social content was associated with an increase in functional connectivity with right superior temporal gyri—but the regional involvement was much less and included portions of precentral gyrus and supplementary motor area. There were no negative associations.

Functional connectivity in left STG was positively associated with social content in left STG whereas right STG connectivity increased with left supplementary motor area.

#### Hypothesis-neutral measures

As expected, there were no significant associations with the scrambled scores. Fluctuations in edge density did not drive corresponding functional connectivity changes in any of the ATL subregions, but did modulate connections between V1 and other visual areas (eg cuneus), suggesting that the observed fluctuations in the ATL subregions are not driven by low-level visual features of the movie (but these features do modulate connections within the visual system). The edge density results are presented in Supplemental Table 3 and Supplemental Fig. 4.

## Discussion

Comprehending the continuous flood of information in natural speech or narratives requires access to conceptual knowledge stored within the semantic system. If the narrative extends beyond a single word or sentence, then simply decoding incoming input is not enough; the semantic system plays a more active role in building and accessing the narrative situation model. In particular, recent evidence suggests that the MTG portion of the ATL hub is active during narrative moments that require greater access to the situation model, such as highly pragmatic content (Thye et al. 2024a). This is observed alongside engagement of regions within the DMN, which is primarily credited with situation model maintenance (Yarkoni et al. 2008; Lee et al. 2020; Sava et al. 2025). Given the known structural connections between anterior MTG and the DMN (Sasaki et al. 2023), this result was interpreted as evidence of endogenous semantic processing, a semantically-driven complement to the functions typically ascribed to the DMN. A natural consequence of this account is that there should be particularly strong functional connections between MTG and the narrative system in DMN. Further, if MTG is part of both the semantic network and the DMN, then these connections should fluctuate with processing demands of the narrative. The aim of the present study was to test these claims by evaluating the extent to which subregions within the graded semantic hubs in bilateral ATL are functionally connected to the DMN and whether these connections are driven by the content within the narrative.

### Functional connectivity

Pairwise comparisons between the functional connectivity maps of the ATL subregions showed evidence of graded changes from ventral (fusiform) to dorsal (superior temporal) portions of the hub. Fusiform and ITG had highly similar connections and both had fewer connections compared to MTG and STG. The sparse functional connections observed for fusiform and ITG may be due (at least in part) to signal drop out (Ojemann et al. 1997), an issue that can be investigated in future studies using a multi-echo sequence optimized for recovering signal in the ventral ATLs (Kundu et al. 2017).

Both MTG and STG had more widespread connections than fusiform or ITG did, including connections to the canonical language network and the DMN. The exception was stronger connections to portions of inferior frontal gyrus and inferior parietal lobule for both fusiform and ITG relative to MTG and STG. Ventral ATL is consistently activated by multimodal semantic processing, so it is unsurprising to observe stronger functional connections to inferior frontal gyrus, a core part of the semantic control network (Jackson 2021), and IPL, an additional semantic hub that is active in semantic tasks (Binder and Desai 2011; Mirman et al. 2017; Jefferies et al. 2019). Relative to MTG, STG had stronger functional connections to the language network extending along STG and including a small portion of inferior frontal gyrus. Conversely, relative to STG, MTG had stronger connections to the DMN, including angular gyri and precuneus. Engagement of the medial frontal portion of the DMN was similar between MTG and STG. Contrasting the connectivity profiles of STG and MTG suggested a graded division along network lines such that STG was more connected to the canonical language network whereas MTG was more connected to DMN regions. This aligns with the graded hub account that suggests STG is related to the auditory modality as a result of receiving inputs from auditory processing regions (Jackson et al. 2016).

Proximal regions tend to have similar connectivity profiles, and this is reflected in the present results among pairs of neighboring regions: anterior fusiform and ITG were similar to one another, as were MTG and STG. However, proximity cannot fully explain the pattern of results: ITG and MTG are also proximal, and are sometimes considered a single functional region called ventrolateral ATL, and yet there were interesting differences between their connectivity profiles.

Connections with DMN were generally stronger for right ATL subregions than for left ATL subregions. This may be because the verbal content of the movies more strongly recruited left ATL regions than right ATL regions (eg Butler et al. 2009; Hoffman and Lambon Ralph 2018). This account is consistent with our broad distinction between endogenous and exogenous semantic cognition and our claim that ATL is involved in both. That is, verbal input drives exogenous semantic cognition, and (because of proximity to the left-lateralized language network) it does so somewhat more strongly for left ATL, thus reducing connections with DMN; the DMN, which drives endogenous semantic cognition, is then more strongly connected to right ATL. This hemispheric asymmetry emerges overall, but, in the present proposal, it is not a fixed property of ATL-DMN connectivity; rather, as we discuss below, it emerges from the dynamic balance between endogenous and exogenous semantic processing demands.

Among ATL subregions, MTG had the strongest functional connections with DMN regions, particularly with angular gyrus and dmPFC. This converges with past evidence that MTG is functionally and structurally connected to the DMN and may serve as part of a distinct dorsal medial subsystem that interacts with the midline core (Andrews-Hanna et al. 2010, 2014; Sasaki et al. 2023). Rather than focusing on the separability or delineation between the default mode and semantic systems, however, a more fruitful avenue for investigating their contributions to cognition may be to study how regions within both networks dynamically interact. Regions and networks often need to be defined for convenience and tractability of analyses, but this can create an exaggerated impression of them as distinct, separable, and independent from one another. Instead of performing highly specialized, within-network functions, regions that fall within both networks may contribute broadly to cognition and support integration between cognitive systems to facilitate processing. Our dynamic connectivity analyses provide evidence for these kind of content-based shifts in connectivity.

### Dynamic connectivity

#### Semantic content

Narrative moments with low semantic content drove an increase in the functional connections between left MTG and core midline DMN regions. This pattern was also present, though much less pronounced, for right MTG. This is consistent with the endogenous semantic processing account claim that when the amount of narrative input is reduced, viewers reflect on and access their situation model (Thye et al. 2023, 2024a, 2024b)—endogenous semantic processing that is supported by stronger connections between the semantic network and the DMN.

High semantic moments require focusing on new information arriving through sensory channels, whereas low semantic moments afford reflection on the broader narrative context. Increasing external task demands are associated with reduced DMN activity and, in the present study, reduced functional connectivity with anterior MTG. This is best illustrated in Fig. 6, which shows the dynamic functional connectivity between MTG and the cluster of core midline DMN regions, and its negative association with semantic content. Moderate to high semantic events (events 5, 17, 293, 324, 328, and 408) were accompanied by a decrease in the MTG-midline DMN connectivity. This is consistent with downregulation of the core DMN during externally-driven, demanding semantic tasks (Humphreys et al. 2015; Shao et al. 2024)*.* Low semantic events (events 85, 97, 211, 220, 402, 412, and 421), conversely, presented little new information but required access to the situation model to fully appreciate their significance. For instance, processing the impact of event 402 (‘*Red on the bus mirroring Brooks when he was released*’) required drawing parallels with event 211 (‘*Brooks on the bus*’). Understanding the significance of Red finding a rock in event 412 required accessing the situation model to remember the instructions Andy gave Red in event 328. These events invite the viewer to reflect on the narrative context, and this drives an increase in the functional connectivity between MTG and midline DMN structures. Note that this negative association between semantic input and functional connectivity with DMN was only observed for MTG and not the other ATL subregions, suggesting this region plays a distinctive role in dynamic coordination of semantic and DMN systems.

Counter to our predictions, we did not observe an increase in engagement of exogenous semantic regions such as anterior fusiform or the broader semantic network when semantic content increased. It is possible that poor signal in ventral ATL partially explains why we did not observe this. Instead, there was a positive association between semantic content and dynamic functional connectivity in a small cluster within middle occipital gyrus, which does not lend itself to straightforward interpretation.

A recent theoretical account argues that the functional role of the DMN is to integrate experiential information into representations of the world—situation models—that lend themselves to semantic, and other, cognitive processing (Ranganath and Ritchey 2012; Yeshurun et al. 2021; Fernandino and Binder 2024). The authors argue that this is true even for isolated words, suggesting a much greater integration between the semantic system and the DMN than previously articulated, and consistent with the observation that DMN regions are routinely recovered in meta-analyses of semantic cognition tasks (Binder et al. 2009). This is compatible with the results observed here, which suggest that rather than having fixed network affiliations, regions (here, specifically anterior MTG) dynamically change connectivity with other portions of the semantic and default mode networks depending on context and processing demands.

#### Social Content

An increase in social content was associated with an increase in connectivity between mid-posterior STG and bilateral anterior MTG and, to a much lesser extent, left anterior STG. This is likely due to the fact that highly social scenes often contain dialogue between two or more characters. As a result, an increase in social content drives increased engagement with the auditory language network. This was more prominent for bilateral MTG compared to STG, possibly because, at a baseline, STG is already more functionally connected to this network compared to MTG.

Interestingly, an increase in social content was not associated with a strengthening in connections between ATL subregions and the social or default mode networks. This is not consistent with a claim that ATL subregions are specialized for social processing. Instead, an increase in social content *decreased* the strength of connections between left MTG and ventromedial prefrontal cortex. This is broadly consistent with the claims made by the endogenous semantic processing account: the MTG subregion is dynamically connected to the DMN and we do not find evidence that this connection is driven by social content. If, instead, the DMN, including MTG, was specialized for processing social content, then connections between MTG and regions within the social network should have positively covaried with social content *or* should not have covaried with semantic content.

We investigated dynamic changes in the functional connections between the semantic and default mode networks using relatively coarse measures of semantic and social content. In prior work (Thye et al. 2024b), we have used these content ratings in activation analyses, which found that highly semantic events engaged a large fronto-temporo-parietal language network, whereas highly social events engaged bilateral superior temporal gyri and right TPJ (among other areas) but not left ATL or MTG. Although our social content measure is likely oversampling moments where more characters are present (and as a result more dialogue), the measures are capturing two dimensions of the narrative content: one that approximates informativeness of the content and another that approximates presence of interpersonal interactions and social reasoning. Although a useful starting point, more nuanced characterisations of the narrative content may better target semantic and DMN interactivity. Low-semantic moments may approximately capture opportunities to engage in endogenous semantic processing; a promising alternative is to identify moments that present minimal new input, but demand active reflection on the situation model. This could be directly manipulated via experimental design or by more focused selection and annotation of naturalistic stimulus such as an audiobook or movie.

## Conclusions

Comprehending an extended narrative (such as a feature-length movie) requires both understanding incoming content (semantic cognition, supported by the hub-and-spoke semantic network, particularly the ATL hub) and maintaining and using a situation model of the narrative (supported by the DMN). The present results suggest the MTG subregion of the ATL plays a particularly important role in coordinating the semantic and DMN systems: it was more functionally connected to the DMN than the other subregions were and these connections were strengthened during narrative moments when the incoming information was limited, providing viewers with a chance to reflect on the content. The latter result is consistent with the distinction between exogenous semantic processing (driven by external input) and endogenous semantic processing (driven by reflecting on the situation model). That is, fluctuations in narrative content produce different demands on the semantic network, which is reflected in dynamic changes in functional connectivity between the semantic network and other neural systems, with the MTG subregion playing a particularly important role in coordinating semantic and DMN systems. More generally, rather than trying to delineate distinct networks and puzzling over their points of overlap, our work suggests that it may be more productive to characterize how neural systems dynamically organize and fluctuate in response to real-world processing demands.

## Supplementary Material

NNDb-FC_Supplemental_Materials_bhaf289

## Data avilability

The raw and preprocessed neuroimaging data are available on OpenNeuro (https://openneuro.org/datasets/ds002837/versions/2.0.0). All analysis code and generated data files are shared on OSF (https://osf.io/76hfv).
