# Supplementary material for: Functional connectivity of semantic and default mode networks during narrative comprehension
Source: Cereb Cortex. 2025 Nov 4;35(11):bhaf289. doi: 10.1093/cercor/bhaf289 (PMC12586323; doi:10.1093/cercor/bhaf289)
Supplement: NNDb-FC_Supplemental_Materials_bhaf289 [file nndb-fc_supplemental_materials_bhaf289.docx]

**Supplementary Materials**

**Functional connectivity of semantic and default mode networks during narrative comprehension**

Melissa Thye^1*^, Junhua Ding^2^, Paul Hoffman^1^, and Daniel Mirman^1^

^1^ Department of Psychology, University of Edinburgh, 7 George Square, Edinburgh EH8 9JZ, UK

^2^ State Key Laboratory of Cognitive Science and Mental Health, Institute of Psychology, Chinese Academy of Sciences, Beijing 100101, China

* Correspondence:

Melissa Thye ([m.thye@ed.ac.uk](mailto:m.thye@ed.ac.uk))

**
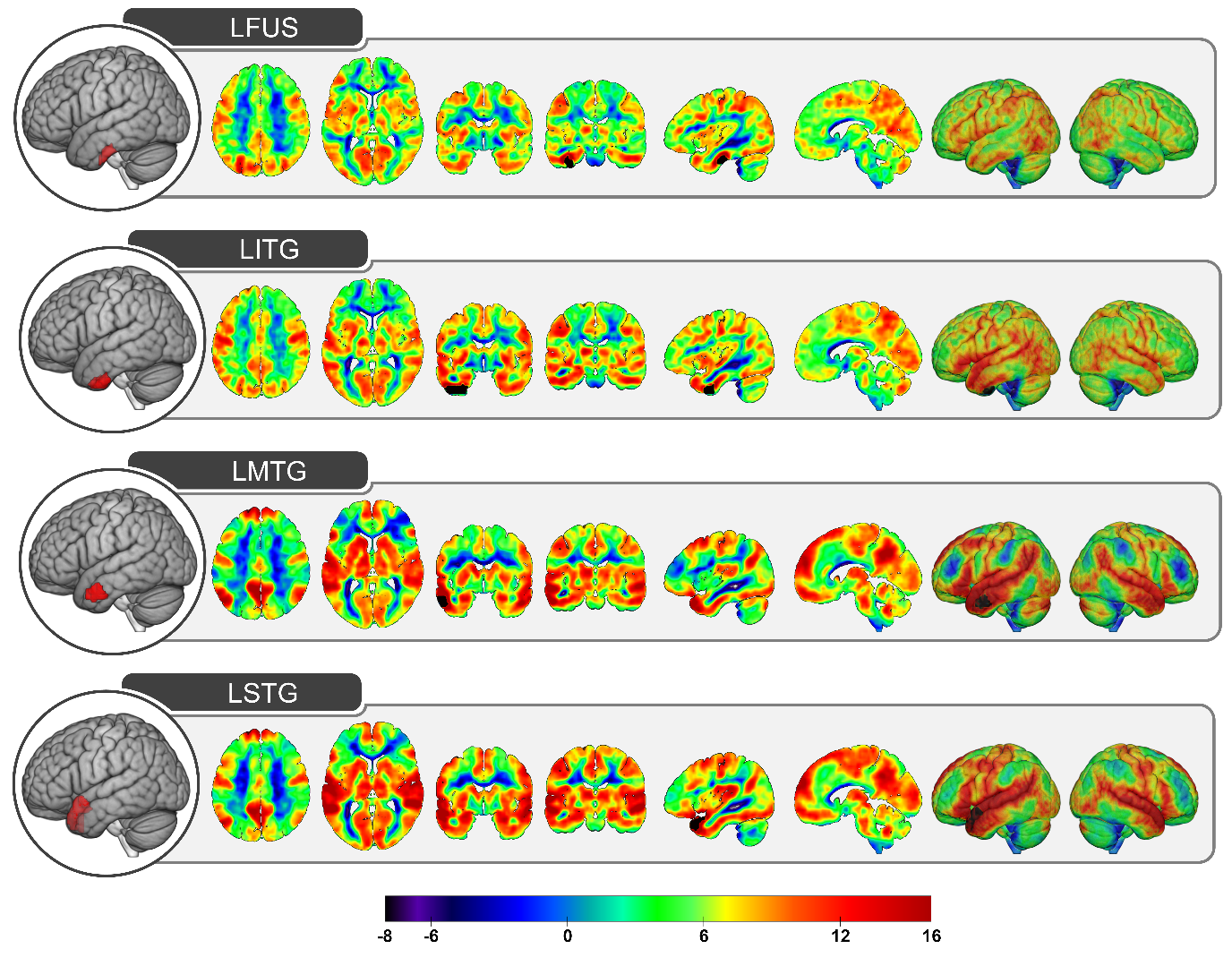
Supplemental Figure 1. Functional connections of left hemisphere ATL subregions.** The *t-*statistic images after conducting a one-sample *t-*test using the normalized functional connectivity maps for each subregion (n=86) as inputs. The ROI used as a seed region is shown in black. LMTG, left middle temporal gyrus; LFUS, left fusiform; LITG, left inferior temporal gyrus; LSTG, left superior temporal gyrus.


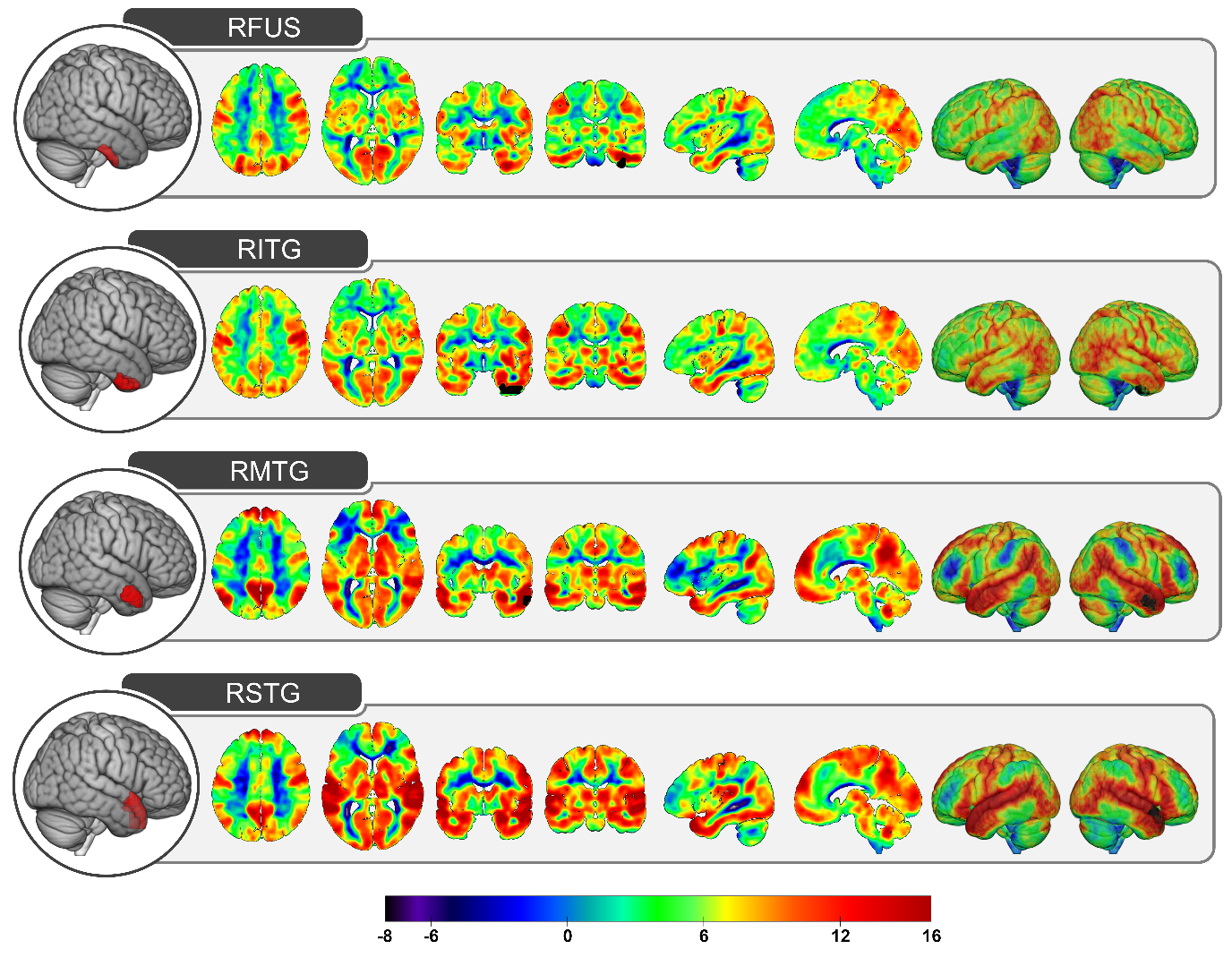
**Supplemental Figure 2.** **Functional connections of right hemisphere ATL subregions.** The *t-*statistic images after conducting a one-sample *t-*test using the normalized functional connectivity maps for each subregion (n=86) as inputs. The ROI used as a seed region is shown in black. RMTG, right middle temporal gyrus; RFUS, right fusiform; RITG, right inferior temporal gyrus; RSTG, right superior temporal gyrus.

**Supplemental Table 1**

*Pairwise Functional Connectivity Comparisons Coordinate Table*

| **Comparison** | **Cluster Size** | **Hem** | **Peak Voxel** | **MNI Coordinates** | | |
| --- | --- | --- | --- | --- | --- | --- |
|  |  |  |  | **x** | **y** | **z** |
| LMTG > LFUS | 9166 | L | Inferior Temporal Gyrus | -55.5 | -1.5 | -31.5 |
|  | 4545 | R | Superior Temporal Gyrus | 52.5 | 16.5 | -25.5 |
|  | 1100 | L | Precuneus | -13.5 | -49.5 | 34.5 |
|  | 971 | L | Middle Cingulate | -1.5 | -16.5 | 40.5 |
|  | 134 | R | Calcarine | 7.5 | -88.5 | 7.5 |
|  | 102 | L | Parahippocampal Gyrus | -19.5 | -4.5 | -13.5 |
|  | 48 | L | Calcarine | -1.5 | -91.5 | -4.5 |
|  | 33 | R | Cerebellum Posterior Lobe | 43.5 | -52.5 | -43.5 |
|  | 32 | L | Cerebellum | -31.5 | -34.5 | -31.5 |
| LFUS > LMTG | 204 | L | Inferior Temporal Gyrus | -37.5 | -22.5 | -28.5 |
|  | 91 | R | Inferior Frontal Gyrus | 49.5 | 43.5 | 13.5 |
|  | 67 | L | Inferior Parietal Lobule | -43.5 | -46.5 | 43.5 |
|  | 62 | L | Inferior Frontal Gyrus (Triangularis) | -46.5 | 40.5 | 13.5 |
|  | 49 | R | Fusiform | 37.5 | -13.5 | -31.5 |
|  | 32 | L | Middle Occipital Gyrus | -25.5 | -64.5 | 40.5 |
| LMTG > LITG | 3599 | L | Superior Medial Frontal Gyrus | -4.5 | 58.5 | 25.5 |
|  | 2617 | L | Fusiform | -55.5 | -1.5 | -28.5 |
|  | 2243 | R | Angular Gyrus | 58.5 | -58.5 | 34.5 |
|  | 1027 | L | Precuneus | -13.5 | -49.5 | 34.5 |
|  | 377 | L | Caudate | -7.5 | 10.5 | 10.5 |
|  | 481 | R | Cerebellum Crus 2 | 28.5 | -85.5 | -34.5 |
|  | 213 | L | Cerebellum Crus 1 | -31.5 | -85.5 | -28.5 |
|  | 173 | R | Caudate | 10.5 | 13.5 | 10.5 |
|  | 94 | R | Cingulate Gyrus | 1.5 | -22.5 | 40.5 |
|  | 56 | L | Cerebellum Crus 1 | -46.5 | -40.5 | -37.5 |
|  | 53 | L | Postcentral Gyrus | -40.5 | -19.5 | 52.5 |
| LITG > LMTG | 319 | L | Inferior Temporal Gyrus | -37.5 | -1.5 | -43.5 |
|  | 195 | R | Fusiform | 31.5 | -7.5 | -40.5 |
|  | 75 | R | Middle Frontal Gyrus | 49.5 | 43.5 | 13.5 |
|  | 70 | L | Inferior Frontal Gyrus (Triangularis) | -49.5 | 43.5 | 13.5 |
|  | 40 | L | Supramarginal Gyrus | -55.5 | -37.5 | 55.5 |
|  | 35 | L | Inferior Parietal Lobule | -40.5 | -46.6 | 40.5 |
| LMTG > LSTG | 560 | L | Inferior Temporal Gyrus | -55.5 | -1.5 | -31.5 |
|  | 372 | L | Angular Gyrus | -58.5 | -58.5 | 34.5 |
|  | 365 | L | Superior Medial Frontal Gyrus | -4.5 | 55.5 | 37.5 |
|  | 301 | L | Precuneus | -10.5 | -55.5 | 34.5 |
|  | 210 | R | Middle Temporal Pole | 52.5 | 13.5 | -31.5 |
|  | 203 | R | Cerebellum Crus 1 | 25.5 | -82.5 | -31.5 |
|  | 192 | L | Cerebellum Crus 2 | -46.5 | -67.5 | -43.5 |
|  | 189 | R | Inferior Parietal Lobule | 49.5 | -58.5 | 49.5 |
|  | 174 | L | Middle Frontal Gyrus | -43.5 | 22.5 | 37.5 |
|  | 104 | R | Superior Medial Frontal Gyrus | 10.5 | 28.5 | 61.5 |
|  | 95 | R | Middle Temporal Gyrus | 67.5 | -19.5 | -10.5 |
|  | 64 | R | Cerebellum Posterior Lobe | 46.5 | -67.5 | -46.5 |
|  | 35 | R | Middle Frontal Gyrus | 43.5 | 22.5 | 46.5 |
|  | 33 | R | Superior Frontal Gyrus | 19.5 | 67.5 | 22.5 |
|  | 32 | L | Middle Frontal Gyrus | -37.5 | 61.5 | -7.5 |
| LSTG > LMTG | 845 | L | Superior Temporal Gyrus | -28.5 | 13.5 | -37.5 |
|  | 251 | R | Superior Temporal Gyrus | 46.5 | 16.5 | -13.5 |
|  | 52 | L | Inferior Frontal Gyrus (Opercularis) | -49.5 | 7.5 | 22.5 |
| LSTG > LITG | 3215 | L | Superior Temporal Gyrus | -28.5 | 13.5 | -37.5 |
|  | 2746 | R | Superior Temporal Pole | 46.5 | 25.5 | -25.5 |
|  | 1700 | R | Superior Frontal Gyrus | 135 | 40.5 | 55.5 |
|  | 692 | R | Precuneus | 1.5 | -52.5 | 34.5 |
|  | 456 | L | Caudate | -7.5 | 4.5 | 13.5 |
|  | 246 | L | Middle Frontal Gyrus | -40.5 | 1.5 | 52.5 |
|  | 225 | L | Middle Cingulate | -4.5 | -7.5 | 49.5 |
|  | 108 | R | Precentral Gyrus | 58.5 | -1.5 | 43.5 |
|  | 100 | R | Cerebellum Crus 1 | 25.5 | -82.5 | -31.5 |
|  | 100 | L | Cerebellum Posterior Lobe | -40.5 | -40.5 | -43.5 |
|  | 87 | R | Cerebellum Posterior Lobe | 4.5 | -52.5 | -43.5 |
|  | 63 | R | Fusiform | 46.5 | -43.5 | -25.5 |
|  | 40 | L | Hippocampus | -25.5 | -22.5 | -13.5 |
|  | 40 | L | Cerebellum Posterior Lobe | -25.5 | -85.5 | -34.5 |
| LIFG > LSTG | 384 | L | Inferior Temporal Gyrus | -46.5 | -4.5 | -40.5 |
|  | 217 | R | Inferior Temporal Gyrus | 52.5 | -10.5 | -34.5 |
|  | 45 | L | Cerebellum Crus 1 | -52.5 | -67.5 | -34.5 |
| LSTG > LFUS | 14702 | L | Middle Temporal Pole | -28.5 | 13.5 | -37.5 |
|  | 819 | L | Cuneus | -1.5 | -67.5 | 25.5 |
|  | 490 | R | Cerebellum Crus 1 | 19.5 | -76.5 | -31.5 |
|  | 291 | L | Calcarine | 04.5 | -94.5 | 7.5 |
|  | 164 | L | Cerebellum Crus 2 | -28.5 | -82.5 | -37.5 |
|  | 87 | L | Fusiform | -43.5 | -43.5 | -19.5 |
|  | 65 | L | Lingual Gyrus | -10.5 | -52.5 | -1.5 |
|  | 31 | L | Inferior Occipital Gyrus | -40.5 | -85.5 | -13.5 |
|  | 30 | R | Cerebellum Posterior Lobe | 28.5 | -64.5 | -49.5 |
| LFUS > LSTG | 217 | L | Inferior Temporal Gyrus | -37.5 | -22.5 | -28.5 |
|  | 45 | R | Fusiform | 37.5 | -13.5 | -31.5 |
| LITG > LFUS | 913 | L | Inferior Temporal Gyrus | -37.5 | -1.5 | -43.5 |
|  | 218 | R | Fusiform | 31.5 | -1.5 | -46.5 |
|  | 180 | R | Superior Temporal Gyrus | 64.5 | -13.5 | -7.5 |
| LFUS > LITG | 157 | L | Inferior Temporal Gyrus | -37.5 | -22.5 | -28.5 |
| RMTG > RFUS | 16940 | R | Middle Temporal Gyrus | 55.5 | 1.5 | -31.5 |
|  | 2411 | L | Precuneus | 1.5 | -61.5 | 37.5 |
|  | 59 | L | Middle Frontal Gyrus | -37.5 | 61.5 | -4.5 |
| RFUS > RMTG | 252 | R | Parahippocampal Gyrus | 34.5 | -22.5 | -28.5 |
|  | 97 | L | Inferior Frontal Gyrus (Triangularis) | -46.5 | 40.5 | 10.5 |
|  | 81 | R | Middle Frontal Gyrus | 46.5 | 43.5 | 7.5 |
|  | 68 | L | Fusiform | -37.5 | -13.5 | -31.5 |
|  | 42 | L | Inferior Parietal Lobule | -43.5 | -46.5 | 43.5 |
|  | 36 | R | Inferior Parietal Lobule | 37.5 | -46.5 | 40.5 |
|  | 34 | R | Supramarginal Gyrus | 61.5 | -34.5 | 49.5 |
|  | 30 | L | Middle Orbitofrontal Gyrus | -28.5 | 37.5 | -10.5 |
| RMTG > RITG | 6805 | R | Middle Temporal Gyrus | 55.5 | 1.5 | -31.5 |
|  | 2170 | L | Angular Gyrus | -55.5 | -61.5 | 25.5 |
|  | 1533 | L | Precuneus | 1.5 | -61.5 | 37.5 |
|  | 681 | L | Caudate | -7.5 | 10.5 | 13.5 |
|  | 349 | L | Cerebellum Crus 2 | -28.5 | -85.5 | -34.5 |
|  | 293 | R | Cerebellum Crus 2 | 28.5 | -85.5 | -34.5 |
|  | 214 | R | Cerebellum Posterior Lobe | 1.5 | -55.5 | -46.5 |
|  | 160 | R | Middle Cingulate | 1.5 | -19.5 | 34.5 |
|  | 132 | R | Cerebellum Anterior Lobe | 28.5 | -31.5 | -31.5 |
|  | 106 | R | Postcentral Gyrus | 52.5 | -19.5 | 49.5 |
|  | 44 | L | Cerebellum Posterior Lobe | -43.5 | -43.5 | -43.5 |
|  | 42 | L | Postcentral Gyrus | -40.5 | -22.5 | 52.5 |
| RITG > RMTG | 427 | R | Inferior Temporal Gyrus | 46.5 | -4.5 | -40.5 |
|  | 174 | L | Inferior Temporal Gyrus | -37.5 | -10.5 | -37.5 |
|  | 105 | R | Middle Frontal Gyrus | 46.5 | 43.5 | 7.5 |
|  | 87 | R | Supramarginal Gyrus | 61.5 | -31.5 | 49.5 |
|  | 83 | L | Inferior Frontal Gyrus (Triangularis) | -49.5 | 43.5 | 10.5 |
|  | 80 | R | Insula | 43.5 | 4.5 | -1.5 |
|  | 74 | L | Inferior Frontal Gyrus (Triangularis) | -46.5 | 13.5 | -4.5 |
|  | 59 | L | Supramarginal Gyrus | -61.5 | -31.5 | 46.5 |
|  | 49 | L | Inferior Parietal Lobule | -40.5 | -46.5 | 40.5 |
| RMTG > RSTG | 735 | R | Superior Medial Frontal Gyrus | 10.5 | 46.5 | 46.5 |
|  | 552 | R | Middle Temporal Gyrus | 55.5 | 1.5 | -31.5 |
|  | 479 | L | Precuneus | -1.5 | -64.5 | 28.5 |
|  | 343 | R | Cerebellum Crus 2 | 28.5 | -85.5 | -40.5 |
|  | 302 | R | Inferior Parietal Lobule | 55.5 | -58.5 | 37.5 |
|  | 317 | L | Angular Gyrus | -52.5 | -67.5 | 34.5 |
|  | 317 | L | Superior Medial Frontal Gyrus | -10.5 | 25.5 | 61.5 |
|  | 289 | L | Middle Temporal Gyrus | -58.5 | 4.5 | -28.5 |
|  | 171 | L | Cerebellum Crus 2 | -34.5 | -82.5 | -40.5 |
|  | 44 | R | Superior Frontal Gyrus | 4.5 | 58.5 | -19.5 |
| RSTG > RMTG | 1936 | R | Superior Temporal Gyrus | 28.5 | 16.5 | -37.5 |
|  | 1399 | L | Superior Temporal Gyrus | -64.5 | -13.5 | 7.5 |
|  | 105 | L | Inferior Frontal Gyrus (Opercularis) | -49.5 | 10.5 | 22.5 |
|  | 91 | R | Inferior Frontal Gyrus (Opercularis) | 52.5 | 13.5 | 25.5 |
|  | 87 | R | Supplementary Motor Area | 1.5 | 4.5 | 64.5 |
|  | 67 | R | Precentral Gyrus | 55.5 | -4.5 | 46.5 |
|  | 44 | L | Inferior Frontal Gyrus (Triangularis) | -34.5 | 22.5 | 10.5 |
|  | 43 | R | Middle Frontal Gyrus | 46.5 | 43.5 | 7.5 |
|  | 32 | R | Middle Cingulate | 7.5 | 16.5 | 37.5 |
|  | 30 | L | Superior Temporal Gyrus | -28.5 | 16.5 | -34.5 |
| RSTG > RITG | 10085 | R | Superior Temporal Gyrus | 28.5 | 16.5 | -37.5 |
|  | 3650 | L | Superior Medial Frontal Gyrus | 1.5 | 58.5 | 28.5 |
|  | 223 | L | Cerebellum Crus 2 | -31.5 | -79.5 | -34.5 |
|  | 161 | L | Cerebellum Posterior Lobe | -7.5 | -58.5 | -40.5 |
|  | 139 | R | Cuneus | 7.5 | -94.5 | 19.5 |
|  | 103 | R | Cerebellum Crus 2 | 28.5 | -82.5 | -34.5 |
|  | 89 | L | Inferior Temporal Gyrus | -46.5 | -43.5 | -19.5 |
|  | 58 | L | Fusiform | -28.5 | -34.5 | -16.5 |
| RITG > RSTG | 499 | R | Inferior Temporal Gyrus | 46.5 | -4.5 | -40.5 |
|  | 171 | L | Inferior Temporal Gyrus | -49.5 | -7.5 | -37.5 |
|  | 48 | R | Cerebellum Crus 1 | 49.5 | -70.5 | -37.5 |
| RSTG > RFUS | 24834 | R | Superior Temporal Gyrus | 55.5 | 7.5 | -16.5 |
|  | 64 | L | Cerebellum Posterior Lobe | -31.5 | -64.5 | -49.5 |
|  | 56 | R | Anterior Cingulate | 1.5 | 22.5 | 22.5 |
|  | 44 | R | Cerebellum Posterior Love | 28.5 | -64.5 | -49.5 |
| RFUS > RSTG | 276 | R | Parahippocampal Gyrus | 34.5 | -22.5 | -28.5 |
|  | 32 | L | Fusiform | -31.5 | -10.5 | -40.5 |
| RITG > RFUS | 844 | R | Inferior Temporal Gyrus | 43.5 | -1.5 | -40.5 |
|  | 140 | L | Middle Temporal Gyrus | -58.5 | -52.5 | 10.5 |
|  | 118 | L | Inferior Temporal Gyrus | -49.5 | 1.5 | -37.5 |
|  | 36 | R | Middle Temporal Gyrus | 55.5 | -64.5 | 13.5 |
| RFUS > RITG | 158 | R | Parahippocampal Gyrus | 34.5 | -22.5 | -28.5 |

*Note.* Hem, hemisphere; L, left; R, right; LMTG, left middle temporal gyrus; LFUS, left fusiform; LITG, left inferior temporal gyrus; LSTG, left superior temporal gyrus; RMTG, right middle temporal gyrus; RFUS, right fusiform; RITG, right inferior temporal gyrus; RSTG, right superior temporal gyrus.

**Supplemental Table 2**

*ATL-DMN Functional Connectivity Pairwise Contrasts*

| **Contrast** | **Estimate** | **SE** | **df** | ***t*-ratio** | ***p*** |
| --- | --- | --- | --- | --- | --- |
| MTG - FUS | 0.065 | 0.003 | 87 | 19.40 | <.001 |
| MTG - ITG | 0.053 | 0.003 | 87 | 18.47 | <.001 |
| MTG - STG | 0.021 | 0.002 | 87 | 10.21 | <.001 |
| FUS - ITG | -0.011 | 0.002 | 87 | -7.04 | <.001 |
| FUS - STG | -0.044 | 0.003 | 87 | -13.70 | <.001 |
| ITG - STG | -0.032 | 0.003 | 87 | -11.40 | <.001 |

*Note.* These contrasts collapse across hemisphere and DMN regions. SE, Standard Error; df, degrees of freedom.


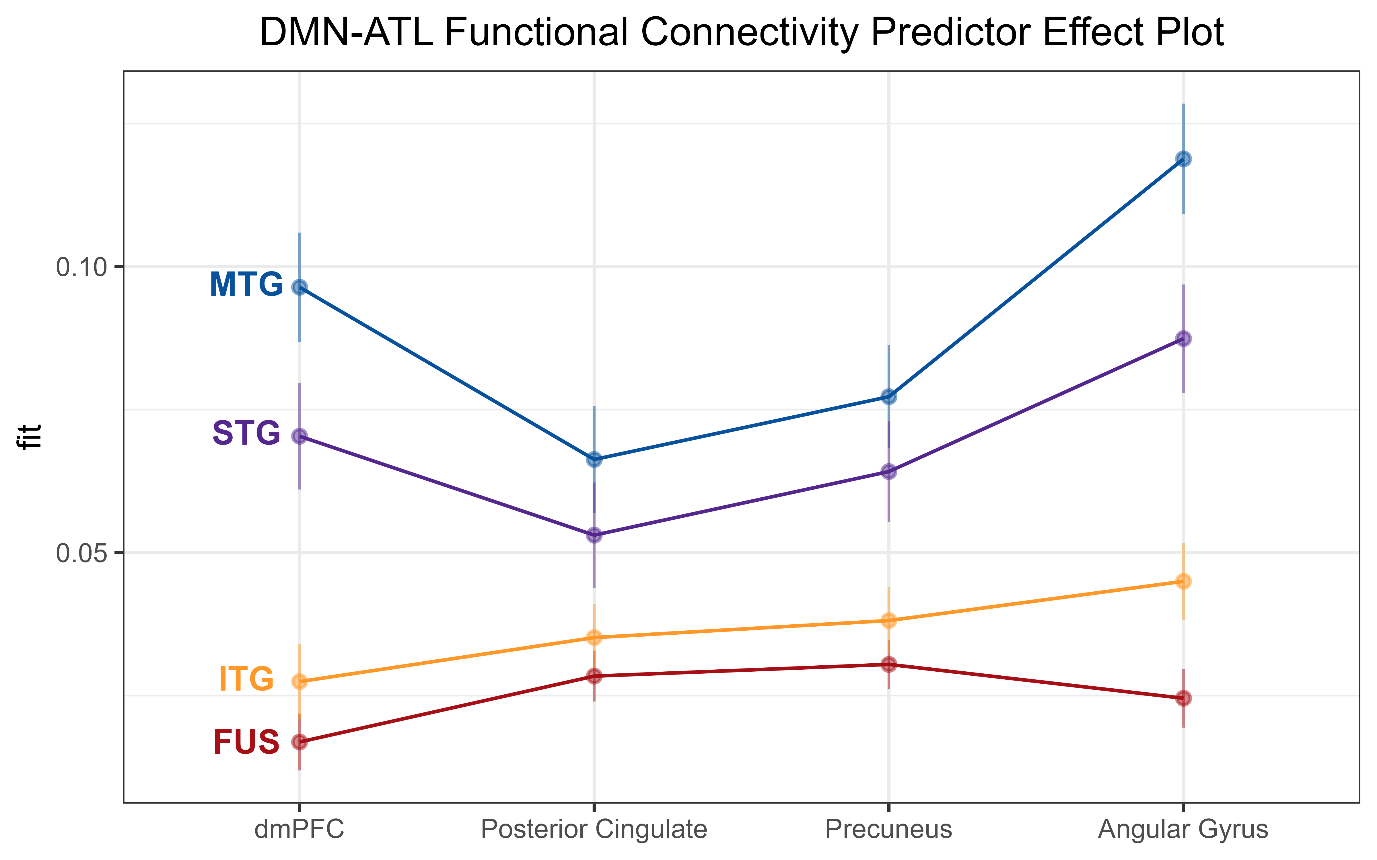


**Supplemental Figure 3.** Predictor effect plot extracted from the mixed effects model showing the model fit values for each DMN region of interest and ATL subregion: FUS, fusiform (red); ITG, inferior temporal gyrus (orange); MTG, middle temporal gyrus (blue); STG, superior temporal gyrus (purple).

**
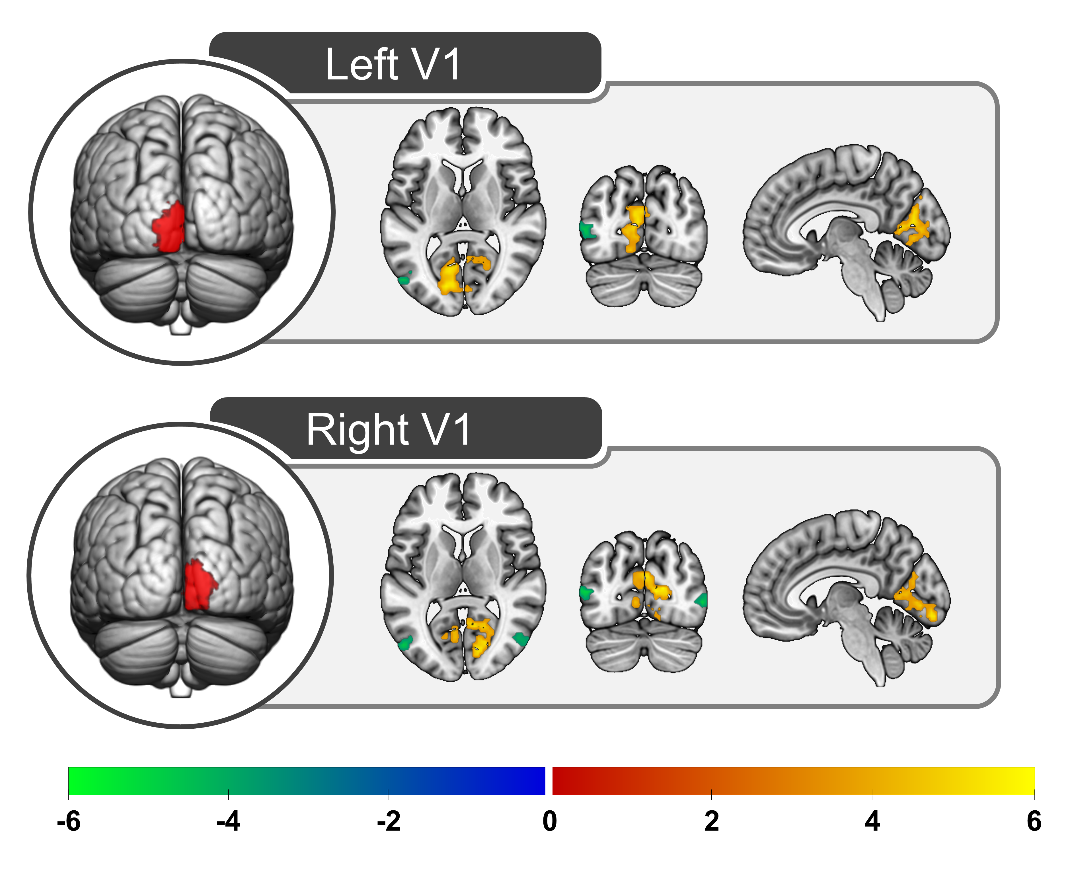
**

**Supplemental Figure 4. Dynamic functional connectivity associations with edge density.** The *t-*statistic images after conducting a one-sample *t-*test using the normalized dynamic functional connectivity maps for each subregion (n=86) as inputs. The strength of negative associations with semantic content is indicated in cool colours, ranging from blue to green. The strength of positive associations with edge density is indicated in warm colours, ranging from red to yellow. Results are displayed on the same slices (axial: 8; coronal: -74; sagittal: -5).

**Supplemental Table 3**

*Edge Density Coordinate Table*

| **Region** | **Association** | **Cluster Size** | **Hem** | **Peak Voxel** | **MNI Coordinates** | | |
| --- | --- | --- | --- | --- | --- | --- | --- |
|  |  |  |  |  | **x** | **y** | **z** |
| Left V1 | positive | 353 | L | Cuneus | -10.5 | -79.5 | 4.5 |
| Left V1 | negative | 96 | L | Middle Temporal Gyrus | -46.5 | -70.5 | 10.5 |
| Right V1 | positive | 528 | R | Cuneus | 13.5 | -79.5 | 13.5 |
|  |  | 45 | R | Parahippocampal Gyrus | 28.5 | -55.5 | -10.5 |
| Right V1 | negative | 63 | L | Middle Temporal Gyrus | -46.5 | -70.5 | 10.5 |
|  |  | 51 | R | Middle Temporal Gyrus | 52.5 | -70.5 | 1.5 |

*Note.* Hem, hemisphere; L, left; R, right.
